# Supplementary material for: Detailed molecular and epigenetic characterization of the pig IPEC-J2 and chicken SL-29 cell lines
Source: iScience. 2023 Feb 20;26(3):106252. doi: 10.1016/j.isci.2023.106252 (PMC10018572; doi:10.1016/j.isci.2023.106252)
Supplement: Data S2. Complete homer output for identified motifs in Chicken SL-29, related to Tables 5 and 6 — Homer motif analysis results for histone modifications H3K4me1, H3K4me3, H3K27ac, enhancers, and ATAC-seq of chicken SL-29 cell line. Parameters for possible false positives is as mentioned earlier for S5. [file mmc3.zip › Data_S2/S6/Chicken_SL_29/peak_files_H3K4me1/homerResults/motif1.similar.html]

motif1

## Information for motif1

T
C
G
A
A
G
C
T
C
T
A
G
C
G
T
A
T
A
C
G
A
C
G
T
G
T
A
C
T
C
G
A
A
G
C
T
G
T
C
A
  
Reverse Opposite:  

C
A
G
T
T
C
G
A
A
G
C
T
A
C
T
G
C
G
T
A
A
T
G
C
C
G
A
T
A
G
T
C
T
C
G
A
A
G
C
T
  

|  |  |
| --- | --- |
| p-value: | 1e-89 |
| log p-value: | -2.064e+02 |
| Information Content per bp: | 1.704 |
| Number of Target Sequences with motif | 1960.0 |
| Percentage of Target Sequences with motif | 3.93% |
| Number of Background Sequences with motif | 1208.9 |
| Percentage of Background Sequences with motif | 2.42% |
| Average Position of motif in Targets | 150.9 +/- 97.9bp |
| Average Position of motif in Background | 149.0 +/- 86.3bp |
| Strand Bias (log2 ratio + to - strand density) | 0.0 |
| Multiplicity (# of sites on avg that occur together) | 1.02 |
| Motif File: | file (matrix) reverse opposite |

### Similar de novo motifs found

|  |  |  |  |  |  |  |  |
| --- | --- | --- | --- | --- | --- | --- | --- |
| Rank | Match Score | Redundant Motif | P-value | log P-value | % of Targets | % of Background | Motif file |
| 1 | 0.957 | T C G A A C G T A C T G C G T A A T C G A C G T A G T C C G T A | 1e-70 | -161.930044 | 6.40% | 4.64% | motif file (matrix) |
| 2 | 0.765 | A C G T C A T G C G T A T A G C A G C T A T G C C G T A A G C T G C A T A C G T A T G C G A T C A G C T | 1e-53 | -123.736556 | 0.39% | 0.10% | motif file (matrix) |
| 3 | 0.778 | T G C A C G A T A T C G C G T A A T C G A G C T G A T C G C T A G A C T G C T A C G A T A C G T G A T C | 1e-27 | -64.137537 | 0.07% | 0.01% | motif file (matrix) |
